# Can We Improve Our Routine Urological Assessment to Exclude Neurogenic Causes for Lower Urinary Tract Dysfunction? ICI‐RS 2024

**DOI:** 10.1002/nau.70028

**Published:** 2025-03-10

**Authors:** Marcus J. Drake, Salvador Arlandis, Marcio A. Averbeck, Enrico Finazzi Agrò, Claire Hentzen, Giovanni Mosiello, Jalesh Panicker, Matthew Smith, Katie Webb

**Affiliations:** ^1^ Department of Surgery and Cancer Imperial College London UK; ^2^ Department of Urology Charing Cross Hospital, Imperial College Healthcare Trust London UK; ^3^ Department of Urology La Fe University and Polytechnic Hospital Valencia Spain; ^4^ Department of Urology Moinhos de Vento Hospital Porto Alegre Brazil; ^5^ Department of Surgical Sciences University of Rome “Tor Vergata” Rome Italy; ^6^ Unit of Urology Policlinico Tor Vergata University Hospital Rome Italy; ^7^ GRC 01, GREEN Group of clinical REsEarch in Neurourology, AP‐HP, Hôpital Tenon Sorbonne University Paris France; ^8^ Division of Neuro‐Urology Bambino Gesù Children Hospital and Research Institute Rome Italy; ^9^ Department of Uro‐Neurology The National Hospital for Neurology and Neurosurgery, Queen Square London UK; ^10^ UCL Queen Square Institute of Neurology, Faculty of Brain Sciences University College London London UK; ^11^ Department of Neurology North Bristol NHS Trust Bristol UK; ^12^ Department of Physiotherapy St Mary's Hospital, Imperial College Healthcare Trust London UK

**Keywords:** autonomic, LUTS, neuro‐urology, Spina bifida, urodynamics

## Abstract

**Aims:**

After presentation with urinary symptoms, an underlying neurological mechanism sometimes emerges subsequently. Increased awareness may bring earlier diagnosis, improving prognosis and outcomes.

**Methods:**

A 2024 International Consultation on Incontinence Research Society think‐tank considered the clinical pathway for identification of an undiagnosed neurological or autonomic contribution precipitating urinary symptoms, and the implications for prognosis.

**Results:**

Alongside adult‐onset neurogenic conditions, potential for missed diagnosis includes congenital and pediatric‐acquired neurogenic conditions, which may become symptomatic during a growth spurt due to spinal cord tethering. Detailed assessment is needed, also considering bowel and sexual dysfunction, with timely referral to neurology to reduce preventable progression of disease. In neurological assessment, control of micturition is often poorly characterized compared with other aspects of spinal cord function and the cranial nerves. Screening tools may be used to identify people who have increased likelihood of particular conditions, but currently available tools are either single‐system or population specific. In addition to the general pelvic examination, the assessment of sacral reflexes and pelvic sensations can suggest a neurological mechanism, though the sensitivity and specificity of the neuro‐urological examination is unknown. Including the results of the neuro‐perineal examination in the urodynamic report may improve the interpretation of the results and potentially support a neurological aetiology.

**Conclusion:**

Future research should consider the value of neuro‐urological examination in diagnosis of occult neurological disease, the development of an occult neurology screening tool/risk scoring based on pelvic organ symptoms, and appropriateness of non‐neurologist practitioners requesting neurological investigations such as MRI scanning.

**Clinical Trial Registration:**

Does not apply.

## 1 Introduction

The possibility of lower urinary tract symptoms (LUTS) or dysfunction (LUTD) representing an early feature of neurological disease is a risk in the LUTS assessment pathway. It means urological assessment and therapy should retain consideration that sometimes an underlying neurological mechanism might emerge subsequently. The International Continence Society (ICS) established a working group which identified the conditions most likely to be responsible [1]. The International Consultation on Incontinence Research Society considered the clinical pathway, discussing how to approach the identification of a neurological contribution in LUTD, and what are the implications for the overall clinical picture.

The ICS consensus categorized the neurological conditions where LUTS can be an early feature as [1];

1. Multiple sclerosis (MS) and related neuro‐inflammatory disorders
2. Multiple system atrophy (MSA)
3. Parkinson's disease
4. Normal pressure hydrocephalus (NPH)
5. Dementia
6. Structural spinal cord conditions

These conditions are predisposed to affect characteristic parts of the nervous system, as set out in Table 1.

**TABLE 1:** Neurological levels affected by disorders that may present initially to a LUTS or urodynamics clinic.

*Neurological level of localization*	*Examples*
**Suprapontine disorders**	**Neurodegenerative synucleinopathies**
	MSA, Parkinson's disease, Dementia with Lewy bodies
	**Inflammatory**
	Multiple sclerosis
	**Vascular**
	Small vessel disease
	**Normal pressure hydrocephalus**
**Supraconal spinal cord disorders**	**Neuroinflammatory**
	Multiple sclerosis
	**Spinal disorder**
	Cervical canal stenosis
	**Dural arteriovenous fistula**
**Conus/cauda equina lesion**	**Spinal disorders**
	Lumbar canal stenosis, Spina bifida occulta
	**Neoplasm**
	Ependymoma and other tumors
	**Vascular**
	Arteriovenous malformation
	**Inflammatory**
	Elsberg syndrome, MOG antibody disease
**Peripheral nerve**	**Autoimmune**
	Vasculitis, Sjogren's syndrome
	**Metabolic**
	Diabetes, Amyloidosis

In LUTS clinics, the imperative is to identify additional atypical features. This could include new onset severe LUTS (excluding infection or malignancy). Alternatively, unusual aspects should be noted, such as emergence of enuresis without chronic retention, which might be a result of a cerebral problem [2, 3]. Furthermore, there may be “suspicious” symptoms reported during history taking, such as numbness, weakness, speech disturbance, gait disturbance, memory loss/cognitive impairment, and autonomic symptoms.

Any such concerns mean that consideration should be given to a more extensive assessment for bowel and sexual dysfunction, as these may develop alongside LUTD in the neurogenic context [1, 4, 5] and a “full house” of pelvic organ involvement may be suggestive of a neurological disorder affecting their common pathways. Despite the risk that pelvic organ problems might indicate an occult neurology, they are often not suspected by healthcare professionals seeing these patients. This is highlighted by the omission of pelvic organ problems for recognition and diagnosis of a range of neurological conditions in recent NICE guidance [6], notably in conditions where pelvic organ symptoms are well established early indications of diseases such as MS and MSA. As pelvic organ problems are common in the general population, it is even more important to distinguish those whose symptoms are being caused by neurological disease, to enable an appropriate and timely referral to an appropriate service (e.g., neurology, neurosurgery or spinal surgery) to reduce the preventable progression of disease, which impacts both the nervous system and the end organs.

Key guidelines [6, 7, 8] instruct the holistic management of bladder, bowel and sexual problems in people with neurological conditions, given that multiple pelvic organ problems are frequently experienced by a single individual. While both healthcare professionals and patients might feel reluctant to discuss sexual problems [9, 10], their evaluation is important for appreciating any neurological contribution and the impact sexual dysfunction has on the patient and their partner.

Screening tools may be used to identify people who have increased likelihood of particular conditions. The currently available tools assessing pelvic organ function are either single‐system specific [11, 12] or population specific [13]. Commonly they quantify symptom severity and impact [14], rather than serving to identify the potential cause of the symptoms i.e. neurological.

### 1.1 Implications of Congenital/Pediatric Conditions (Occult or Identified)

Neurogenic causes of LUTD in children have been mostly related to spina bifida and other spinal dysraphism (SD). Myelomeningocele, the so‐called open spina bifida, is easily recognized at birth and prenatally. Other forms of SD (“closed spina bifida”) are often only recognized later in childhood, due to a skin change in the lumbo‐sacral area, or the presence of neurological, urological, and/or orthopedic symptoms [15].

The potential for a missed diagnosis of a congenital or pediatric‐acquired neurogenic LUTD is potentially under‐estimated. SD includes a heterogeneous group of neural tube defects, related to a large spectrum of clinical entities. The range covers severe forms, diagnosed in childhood, to silent forms discovered incidentally during and after puberty, or even in adulthood. SD may become evident only with the emergence of symptoms from cord tethering [16]. Tethered cord syndrome is a stretch‐induced disorder of function of the lumbosacral spinal cord due to excessive tension. Symptoms may include LUTS, scoliosis, clubfoot, back pain, constipation, gait disturbances, and so on. In adolescents and young adults, symptoms from cord tethering are often precipitated by rapid growth in height and exacerbated by puberty, in association with prostate enlargement or urethral oestrogenization [17]. In some cases, patients have been investigated previously inappropriately. SD has been reported in up to 46% in patients with anorectal malformation (ARM). Neonates with ARM are still mainly screened with spinal ultrasound (US), yet this cannot reliably detect all forms of SD, especially after the third month of life [18]. Magnetic Resonance imaging (MRI) is sensitive for SD, but should evaluate all spine segments and the brain. Some authors suggest performing the MRI in the prone position to increase specificity [19]. In other cases mistakes are related to use of an inappropriate classification, such as low‐ versus high‐risk of tethering, which can cause controversy related to diagnosis and treatment options [20]. A new classification modality, including prenatal parameters, may reduce the risk of this issue [21, 22]. Uro‐neurological assessment and imaging (spine and brain) are important to exclude congenital neurological causes of LUTD, because they may present with urinary symptoms as the sole initial symptom [23].

Another risk area is failure to recognize the implication of an aspect of past medical history, specifically that healthcare professionals may not appreciate that a condition can be associated with neurogenic LUTD. This includes conditions that have improved substantially from a severe initial problem (e.g., transverse myelitis) or have remained static. Examples include children with so‐called “special needs” and intellectually and/or motor disabled people (notably Cerebral Palsy (CP), Down Syndrome, Rett Syndrome, Angelman Syndrome, Fragile‐X Syndrome, Williams Beuren Syndrome, X‐linked adrenoleukodystrophy), and those with rare diseases (e.g., Guillain–Barré Syndrome, Acute Disseminated Encephalomyelitis, Meningitis‐Retention Syndrome). In CP, LUTS are present in about a third of children, in proportion to the severity of motor and/or cognitive disabilities. Many adult CP patients also present in the second or third decade of life with LUTD, particularly with urinary retention. LUTS can start in the pediatric age range, but may be missed if parents and physicians are mainly focused on physical rehabilitation, or management of respiratory or nutritional concerns. Urinary incontinence might even be considered “normal” in some conditions, and hence not be evaluated. This means upper urinary tract evaluation may not be done, resulting in young adults presenting with avoidable renal impairment at first urological assessment [24].

Overall, missed pediatric NLUTD, or progression of a pediatric condition, is an important consideration in adolescents and young adults. NLUTD must be excluded in the presence of; recurrent or persistent incontinence, complicated incontinence (with pain and/or haematuria), recurrent UTIs, relapse of vesicoureteral reflux or presence of renal scarring, and LUTD with upper urinary tract impairment not responding to treatment.

### 1.2 LUTS Clinic Assessments Preceding Referral to Neurology

In the neurological assessment, control of micturition is not as well characterized as other aspects of spinal cord function and the cranial nerves. A comprehensive history should focus not only on LUTS, but should also assess bowel and sexual functions. The state of the rectum significantly influences the sensations of bladder filling [25]. Neurogenic LUTD and bowel dysfunction, overlap substantially, and may alter congruently over time [26]. Available data on neurological patients suggest that neurogenic bowel dysfunction with chronic constipation may mechanically contribute to LUTD. Chronic constipation and stool impaction may develop over many years, and affected people are often subjectively unaware. The physical examination is crucial for patients with suspected or confirmed NLUTD [27]. Testing is necessary for all urogenital sensations and reflexes. Moreover, comprehensive testing of the pelvic floor and anal sphincter must be carried out. Depicting discrete regions of sensory impairment facilitates a more precise localization of the neurological lesion.

Pelvic examination is mandatory in patients reporting LUTS. In addition to the general pelvic examination, the assessment of sacral reflexes and pelvic sensations can suggest a neurological mechanism, though the sensitivity and specificity of the neuro‐urological examination is unknown. No normative data exist on sacral sensations as age, hormonal status, and menopause can impact sensations on the genitalia [28]. However, the absence of touch or prick sensations, or an asymmetry between sides, should be considered as abnormal. Sacral reflexes include bulbocavernosus and anal, and the absence of both reflexes is suspicious for neurological dysfunction. However, 20% of healthy women do not have bulbocavernosus reflexes [29]. Moreover, the impact of conditions such as fecal impaction or bladder fullness on the facility to elicit these reflexes is unknown.

Assessing the voluntary pelvic floor contraction is part of the physical examination, but inability to contract the pelvic floor muscles is common in women, regardless of neurological status. A reduced anal tone can imply various mechanisms, including peripheral neurological dysfunction. The value of increased anal tone is unknown, especially in young people or in a context of anxiety. A limitation is the inter‐examiner agreement of the assessment of anal tone with the DRESS score, which is poor to moderate, especially in beginner examiners [30]. Overall, the physical examination can support or go against a primary neurological hypothesis, but it is not primarily diagnostic.

### 1.3 Urodynamics Where the Mechanism May Relate to an Undiagnosed Neurological Condition

Including the results of the neuro‐perineal examination in the urodynamic report may improve the interpretation of the results and potentially support a neurological etiology. Thus, specific training of healthcare professionals for neuro‐urological examination in each potential context is likely to be valuable. In addition, it can identify issues that might influence assessments, for example by highlighting whether the rectum is empty, as it needs to be for both reliable abdominal pressure recording during urodynamic testing and because rectal loading may impair detrusor contractility.

Urodynamic tests can reveal abnormalities which may strongly suggest a neurological lesion. Impairment of the proximal urethral sphincter mechanism in men (open bladder neck) [31] is important. Provided there is no history of a relevant surgical procedure (bladder neck operation, retroperitoneal dissection or sympathectomy), this may suggest a lesion affecting the intermediolateral nucleus in the thoracic cord, or the pathways supplying it. Partial detrusor external sphincter dyssynergia [32] may suggest a lesion in the suprasacral pathways, provided dysfunctional voiding is excluded. Abnormalities of the sphincter EMG suggest a neurological mechanism [33], though this is evaluated in comparatively few centers.

Other findings, such as detrusor overactivity (DO), absence of sensation or detrusor areflexia may occur without a neurological disease, so are not diagnostic [33]. The International Consultation on Incontinence (ICI) 2017 concludes that studies have not shown significant differences in the patterns or characteristics of DO based on whether the cause is neurogenic or idiopathic (level of evidence: 2). Therefore, the committee recommends (grade C) that neither the cause (neurogenic or idiopathic) nor the severity of DO should be diagnosed solely based on urodynamic parameters. Nonetheless, clinical experience indicates that some refractory OAB patients develop neurogenic conditions during follow‐up (e.g., dementia or MSA).

For patients with reduced detrusor compliance (raised bladder filling pressure), suspicion should be high for a neurological mechanism. It can be quantified in terms of the compliance, and, if applicable, the detrusor leak point pressure (DLPP), which is the lowest detrusor pressure at which urine leakage occurs in the absence of either a detrusor contraction or increased abdominal pressure [2]. DLPP has been historically considered a predictor of upper urinary tract (UUT) deterioration [34], with a DLPP > 40 cmH_2_O associated with UUT deterioration. Detrusor overactivity leak point pressure (DOLPP) is defined as the lowest detrusor pressure rise with detrusor overactivity at which urine leakage first occurs in the absence of a voluntary detrusor contraction or increased abdominal pressure. This is a complex parameter, as urethral closure is potentially confounded by other influences, notably the obstetric history and its secondary factors such as stress urinary incontinence in women [35]. If such factors are absent, a low DOLPP may suggest impaired sphincter function. The presence of DO and a weak sphincter with no other explanation may be suggestive of a neurological cause.

### 1.4 What Are the Harms That Can Arise From Missing a Neurological Diagnosis in a Patient Presenting to LUTS Clinic?

Healthcare professionals in LUTS or urodynamics clinics can find themselves in a vital position where they have the chance to raise the suspicion of a neurological disorder and potentially improve outcomes beyond simply managing symptoms of LUTS. In the majority of cases, earlier diagnosis of a neurological condition leads to some form of improved outcome. For structural spinal disorders such as lumbo‐sacral stenosis, or spinal dural arteriovenous fistula, diagnosis and subsequent intervention is associated with improved outcomes [36, 37], but may be difficult to diagnose at times due to a lack of other neurological symptoms. LUTS are common in multiple sclerosis and may represent the sole symptom of a spinal inflammatory plaque. In a patient who has an unexplained picture of OAB, early investigation leading to diagnosis can improve time to receipt of the wealth of disease modifying therapies now available, with a more aggressive immunosuppressive approach increasingly taken by neurologists [38]. Other conditions that may present early with LUTS may not have disease modifying therapy, for example MSA. However, timely diagnosis still remains important. Diagnosis can lead to appropriate symptomatic treatment being given, and in the case of progressive conditions, can help patients ensure that they prioritize their time whilst they remain mobile and independent. Hence, while studies exploring the impact of diagnosing neurological conditions following early urological assessment have yet to be performed, prompt referral should be emphasized.

### 1.5 Should a Urologist or Urogynaecologist Request Neuroimaging?

The utility of the non‐neurologist proceeding with neuroimaging investigations where a neurological condition is suspected, rather than waiting for assessment by a neurologist, has not been investigated. The ICS consensus did not support non‐specialists leading on the selection of neurological imaging tests, for various reasons including risk of missing important regions to be scanned [1]. Nonetheless, in cases where isolated NLUTD is found, spinal cord MRI is likely to be the first investigation requested by a neurologist, and pre‐empting this could improve efficiency of care pathways. An additional benefit of this approach is where treatable structural cord problems (e.g., disc disease) may be discovered on the MRI, allowing the patient to be referred straight to a spinal surgeon rather than via a neurologist. One issue is familiarity with this investigation for non‐neurologists and, in particular, interpreting and communicating findings such as incidental degenerative disease.

A natural extension of the above question is whether MRI imaging of the brain may be appropriate for a urologist or uro‐gynecologist to organize pre‐emptively. Due to the neuroanatomy of the brain, finding a structural lesion causing isolated LUTS without other neurological symptoms is unlikely. Additionally imaging of the brain more commonly uncovers incidental findings [39], such as meningiomas, aneurysms and small vessel disease, which necessitates resources for further investigation and causes distress to patients. Unfortunately, there may be a risk that the most suitable MRI scan will not be requested (e.g., a non‐neurologist may put in an imaging request which fails to include an important part of the neuraxis). Furthermore, radiologists are specialized, and in some health systems they are linked to particular services. Hence, a urological request may go to a urological radiologist, which may result in difficulties deciding on the most suitable scanning protocol. There is also a challenge in reporting, since incidental findings might be reported as significant if the radiologist has comparatively limited experience of reviewing neurological MRI scans. Accordingly, it is appropriate to bear in mind the local policy related to radiology support.

Overall, the clinical utility and health economic cost of opening out specialist testing to healthcare professionals who are not specialists in the area are far from clear. Having a multidisciplinary team with access to specialist neurological advice could help facilitate non‐neurologists in managing these issues, aiding in test selection and subsequent interpretation, improving the efficiency of clinical pathways.

An important factor is to recognize that sometimes urinary tract dysfunction can reflect a condition which is potentially managed by neurosurgery, in which case spinal cord imaging could be undertaken to help direct onward specialist referral to the appropriate department. Nonetheless, such conditions are commonly managed jointly by neurology and neurosurgery, so discussion with colleagues is appropriate before placing an imaging request.

### 1.6 Disorders of the Autonomic Nervous System

Autonomic disorders were not considered in the ICS consensus statement, yet the dominant role of the parasympathetic and sympathetic nervous systems in lower urinary tract function means such disorders have a high chance of eliciting LUTS and LUTD. This can be primary autonomic dysfunction, or a significant autonomic component of another neurological condition. Such disorders can present as a myriad of symptoms depending upon the organ affected. Patients can present to an eye specialist for dry eyes and blurred vision, a cardiologist for palpitations, orthostatic light‐headedness or syncope, a musculoskeletal specialist for orthostatic pain over the back of the neck extending to both shoulders in the distribution of a coat hanger, a gastroenterologist for abdominal bloating, and altered bowel movements ranging between constipation and diarrhea, or a neurologist when autonomic failure is associated with neurological symptoms such as imbalance, falls and limb sensorimotor symptoms. Patients present to a urologist or gynecologist when the autonomic innervation to the LUT and genitalia is affected, presenting with storage and voiding symptoms, urinary retention, erectile dysfunction, sexual arousal disorders and/or poor ejaculation. Autonomic disorders can be grouped according to the site of neurological localization:

1. Central disorders, for example α‐synucleinopathy neurodegenerative disorders (Parkinson's disease, MSA, Dementia with Lewy bodies).
2. Peripheral disorders.

a. Pure autonomic failure, for example autoimmune autonomic ganglionopathy, in which high titres of antibodies against the neuronal ganglionic nicotinic acetylcholine receptor (AChR) are identified.
b. Small fiber neuropathies, for example due to diabetes, amyloidosis (Amyloid light chain [AL] or Amyloid transthyretin protein [ATTR] aggregation), or autoimmune vasculitis (e.g., Sjogren's syndrome).
c. Hereditary sensory and autonomic neuropathies.

Urogenital symptoms are reported in all of these disorders. In general, it is not known how often the initial medical presentation is seen in a LUTS clinic. MSA is well‐recognized for this, likely from early sacral spinal cord involvement associated with erectile dysfunction, detrusor underactivity [40] or OAB symptoms [41] during a prodromal pre‐motor phase of the disease [42]. MSA can present initially with urinary retention.

History and examination should identify whether the eyes and/or mouth are dry, as a consequence of impaired glandular secretions including the salivary glands. The tendency to cold extremities, with or without blue discolouration, reflects impairment of peripheral circulatory regulation. Blood pressure should be checked when lying down and 3 min after standing up. Orthostatic hypotension is diagnosed by a sustained reduction in systolic blood pressure of at least 20 mmHg or diastolic blood pressure of 10 mmHg within 3 min of standing after being supine for 5 min. Autonomic dysfunction can also affect swallowing, stomach emptying, constipation, sexual function. In severe cases it may affect hearing (regulation of sound sensitivity) and light sensitivity (control of the iris).

### 1.7 Research Questions

The ICIRS think tank reviewed several questions and finalized a shortlist of the following priority research needs;

- What is the value (sensitivity, specificity) of neuro‐urological examination in the diagnosis of occult neurological disease?
- Would a screening tool for potential occult neurology based on pelvic organ symptoms improve identification of symptoms as neurogenic in nature?
- Can a risk score in predetermined suspect clinical situations be built to assess the probability of neurological etiology of LUTS, based on clinical examination and urodynamic findings?
- Can neuro‐urological examination be included as part of a urodynamic report to improve the interpretation. How should healthcare professionals be trained to perform and report the results of such examinations?
- Is it appropriate for a healthcare professional who is not a neurologist to request investigations which are primarily neurological (including MRI scanning of part of the neuraxis)?

Each of the above questions would require retrospective scrutiny of the initial evaluations undertaken once the final diagnosis is known, hence will be reliant on accurate long‐term data.

### 1.8 Conclusions

Healthcare professionals should maintain a high index of suspicion for a neurological condition contributing to LUTS. If an underlying neurological mechanism is suspected, detailed assessment should consider wider features, including bowel and sexual dysfunction. Timely referral to neurology might reduce preventable progression of disease. Research is needed to evaluate neuro‐urological examination for diagnosis of occult neurological disease and nonspecialist requesting of neurological investigations, and to develop a screening tool or risk scoring.

## Author Contributions

All authors contributed to the conceptualization, writing, and approval of the manuscript.

## Data Availability

The authors have nothing to report.
